# PDlim2 Selectively Interacts with the PDZ Binding Motif of Highly Pathogenic Avian H5N1 Influenza A Virus NS1

**DOI:** 10.1371/journal.pone.0019511

**Published:** 2011-05-23

**Authors:** Jia Yu, Xin Li, Yu Wang, Bo Li, Hongyue Li, Yapeng Li, Weihong Zhou, Cuizhu Zhang, Yingying Wang, Zihe Rao, Mark Bartlam, Youjia Cao

**Affiliations:** 1 Tianjin Key Laboratory of Protein Science, College of Life Sciences, Nankai University, Tianjin, China; 2 Key Laboratory of Pollution Processes and Environmental Criteria (Ministry of Education), College of Environmental Science and Engineering, Nankai University, Tianjin, China; University of Hong Kong, Hong Kong

## Abstract

The multi-functional NS1 protein of influenza A virus is a viral virulence determining factor. The last four residues at the C-terminus of NS1 constitute a type I PDZ domain binding motif (PBM). Avian influenza viruses currently in circulation carry an NS1 PBM with consensus sequence ESEV, whereas human influenza viruses bear an NS1 PBM with consensus sequence RSKV or RSEV. The PBM sequence of the influenza A virus NS1 is reported to contribute to high viral pathogenicity in animal studies. Here, we report the identification of PDlim2 as a novel binding target of the highly pathogenic avian influenza virus H5N1 strain with an NS1 PBM of ESEV (A/Chicken/Henan/12/2004/H5N1, HN12-NS1) by yeast two-hybrid screening. The interaction was confirmed by *in vitro* GST pull-down assays, as well as by *in vivo* mammalian two-hybrid assays and bimolecular fluorescence complementation assays. The binding was also confirmed to be mediated by the interaction of the PDlim2 PDZ domain with the NS1 PBM motif. Interestingly, our assays showed that PDlim2 bound specifically with HN12-NS1, but exhibited no binding to NS1 from a human influenza H1N1 virus bearing an RSEV PBM (A/Puerto Rico/8/34/H1N1, PR8-NS1). A crystal structure of the PDlim2 PDZ domain fused with the C-terminal hexapeptide from HN12-NS1, together with GST pull-down assays on PDlim2 mutants, reveals that residues Arg16 and Lys31 of PDlim2 are critical for the binding between PDlim2 and HN12-NS1. The identification of a selective binding target of HN12-NS1 (ESEV), but not PR8-NS1 (RSEV), enables us to propose a structural mechanism for the interaction between NS1 PBM and PDlim2 or other PDZ-containing proteins.

## Introduction

The influenza A virus has long posed a threat to human health. The avian influenza H5N1 virus exhibits significantly higher pathogenicity than other strains, with nearly 60% lethality in human infections (WHO, 2010). Previous studies have shown that pigs infected with a recombinant human H1N1 influenza virus in which the NS1 gene is replaced with that from the H5N1 strain experienced significantly more serious fever, weight loss and viremia than those infected by the wild-type human influenza H1N1 virus [1]. The NS1 protein was consequently identified as an important factor determining the virulence of the influenza virus. As a multifunctional protein, NS1 predominantly impacts host immune response during viral infection by reducing the induction of IFN-β [2], [3], inhibiting the RNA sensor activity of human retinoic acid-inducible gene product I (RIG-I) [4], suppressing the dsRNA dependent protein kinase (PKR) induced protein synthesis termination [5] and limiting the activation of 2′-5′-oligoadenylate synthase (OAS) [6], as well as disrupting interferon signaling by reducing the tyrosine phosphorylation of STAT proteins [7]. Other studies have demonstrated that NS1 is able to integrate with phosphatidylinositol-3-kinase (PI3K) and prevent apoptosis during infection [8], [9].

Obenauer and colleagues noted that the C-terminal four amino acids of NS1 constitute a type I PDZ binding motif (PBM) [10], [11]. PDZ (PSD-95/Dlg-A/ZO-1) domains contain approximately 80 amino acids and act to mediate interaction with other proteins [12]. Therefore, PDZ-containing proteins are widely considered to act as scaffold proteins. Due to their affinity to the cytoskeleton, they are thought to regulate cell migration, adhesion and polarity depending on the cellular localization of the protein [13]. In a previous study, 30 human PDZ-domain-containing proteins were identified to be able to bind to a PBM with the sequence ESEV, which is commonly found in the NS1 protein from avian influenza viruses, including those with the H5N1 subtype [10]. A large scale analysis demonstrated that 92% of NS1 proteins sourced from avian influenza viruses contain a PBM bearing the sequence ESEV, while NS1 proteins sourced from human influenza viruses mostly bear PBM with the sequence RSKV or RSEV. Only about 8% of human influenza virus NS1 proteins bear a H5N1-like PBM (with sequence EPEV or ESEV), and these viruses are linked to high-mortality outbreaks of influenza [10]. A subsequent study showed that substituting the human NS1 PBM with a PBM derived from highly pathogenic avian influenza (HPAI) H5N1 viruses (either ESEV or EPEV) did not affect the growth of virus but significantly increased its virulence and pathogenicity in mice [14]. Therefore, the NS1 PBM is suspected to be a virulence-determining ligand in a host- and species-dependent manner [15], [16].

One of the functions of the ESEV PBM was recently discovered to be in apoptosis limitation during viral infection via direct interaction with the cellular PDZ-containing protein Scribble [17]. The relationship between the precise amino acid composition in the PBM and its binding ability with certain PDZ-containing proteins has been reported [18]. However, the detailed structural basis for their interaction remains to be identified. In this study, we identified PDlim2, another PDZ-containing protein, as a binding target of NS1 encoded by a highly pathogenic H5N1 avian influenza virus, A/Chicken/Henan/12/2004 (HN12-NS1), via the PDZ-PBM interaction *in vitro* and at the cell level. We found that the PDZ domain of PDlim2 binds specifically to the C-terminal PBM sequence ESEV of HN12-NS1 but not to the NS1 protein from an H1N1 strain, A/Puerto Rico/8/34 (PR8-NS1), which has a corresponding PBM sequence of RSEV. Analysis of the crystal structure of the PDlim2 PDZ domain in complex with the C-terminal hexapeptide of HN12-NS1 provides a molecular basis to explain the binding mechanism and selectivity between the two proteins, and indicates why PDlim2 binds to HN12-NS1 (ESEV) but barely to PR8-NS1 (RSEV). This work, although preliminary, provides a structural basis for the binding selectivity of PDZ-containing proteins to the ESEV-terminal NS1 of the highly pathogenic H5N1 strain, and thus leads to further understanding of the mechanism for the PDZ-PBM interaction.

## Materials and Methods

### Cells, Plasmids and Antibodies

HeLa (ATCC:CCL-2) cells were cultured in Dulbecco's Modified Eagle Medium (DMEM) supplemented with 10% fetal bovine serum. The plasmid containing the coding sequence of NS1 of A/Puerto Rico/8/34 /H1N1 (Accession No.:CY009448, GenBank), pCAGGS-P7-PR8-NS1, and total RNA of H5N1 influenza virus (A/Chicken/Henan/12/2004/H5N1), Accession No.:AY950260, GenBank) were kindly provided by Z. Chen (College of Life Sciences, Huhan Normal University, China). For GST pull-down assays, cDNA encoding NS1s and His-tagged PDlim2 (Accession No.: AAH21556.1, GenBank) were respectively inserted into pGEX-4T-1 (GE) and pET-28a (Novagen). PDlim2 mutations were derived from the constructed wild type PDlim2 plasmid using the Easy Mutagenesis System kit (Transgen). For the two hybridization assays, the commercial vectors, pGBT9 and pACT2 (Clontech), and pACT, pBind and pG5*luc* (Promega) were constructed according to the manufacturer's instructions. For purification and crystallization, a DNA fragment encoding 89 amino acid residues, including the PDlim2 PDZ domain (residues 1–83) fused with a C-terminal hexapeptide extension (sequence: TIESEV) corresponding to residues 220–225 of HN12-NS1, was constructed into pGEX-6P-1 (GE Healthcare) [19], [20]. Anti-GST (#2622) and anti-His (sc8036) antibodies were purchased from Cell Signaling and Santa Cruz, respectively; Anti-HA (H9658) and anti-Flag (F1804) antibodies were brought from Sigma; Anti-STAT1 (AHO0832) and anti-phospho-STAT1 (33–3400) were from Invitrogen.

### Yeast Two-Hybrid Screening

In yeast two-hybrid assays, pGBT9-HN12-NS1 and pACT2 into which a human spleen library gene was inserted were transfected into yeast (*Y190*). After auxotrophic and X-gal double selection, the plasmids of positive clones were extracted and sequenced following the protocol in the commercialized kit (Clontech).

### Mammalian Two-Hybrid Assays

pACT-NS1, pBind-PDlim2 and pG5*luc* were co-transfected into HeLa cells with a molar ratio of 1∶1∶1 as suggested (Promega), with Polyethylenimine (PBI) (1mg/ml) [21], [22]. After 48 h, cells were harvested and lysed, and the firefly and Renilla luciferase activities were quantified by the Dual-Luciferase Reporter Assay (Promega). The relative luciferase activity was indicated by the ratio of firefly to Renilla florescence intensities.

### GST Pull-Down Assays

GST-tagged NS1 or associated mutants and His-tagged PDlim2 or associated mutants were expressed in *Escherichia coli* BL21 (DE3), respectively. The bacterial lysates containing NS1 were incubated with Glutathione Sepharose 4B (GE) beads with gentle rolling in lysis buffer (50mM Tris-Cl, 150mM NaCl, 0.5% NP-40 and protease inhibitor cocktail) at 4°C for 1 h. After discarding the supernatant, the lysate containing His-PDlim2 was added to the beads and incubated for 2 h with gentle rolling. The resultant beads were washed with the lysis buffer four times and the eluants were subjected to SDS-PAGE and Western blot analysis with antibodies against His-tag and GST respectively.

### Bimolecular Fluorescence Complementation (BiFC) Assays

The BiFC method was adopted to investigate protein binding in HeLa cells [23]. PDlim2 proteins were fused to the carboxyl-terminal fragment (YC, aa155–238) of yellow fluorescence protein (YFP). The HN12-NS1wt or HN12-NS1ΔPBM coding region was fused with the N-terminal fragment (YN, aa1–154) of YFP [23]. YC-PDlim2 and the YN-HN12-NS1wt or YN-HN12-NS1ΔPBM proteins were co-transfected into HeLa cells grown on glass cover slips using the PBI method as described above. 24 hours after transfection, cell culture dishes were transferred to room temperature for 4 hours and stained with 4′,6-diamidino-2-phenylindole (DAPI) prior to microscopic analysis. The fluorescence emission was examined in living cells by confocal microscopy and images were processed using an Olympus Fluoview FV1000. The YFP fluorescence of living cells was also subjected to FACS analysis with an excitation wavelength at 488 nm and emission wavelength at 513 nm.

### Protein Purification and Crystallization

The GST-tagged PDlim2 PDZ domain fused with a C-terminal hexapeptide extension (sequence: TIESEV) corresponding to residues 220–225 of HN12-NS1 was expressed in *E. coli* BL21 (DE3). The harvested bacterial cells were lysed by sonication and a standard GST affinity purification procedure was performed using glutathione agarose beads. The GST-tag was removed with PreScission Protease, and the resultant fusion protein was further purified with a Superdex 200 10/300 GL gel-filtration column (GE Healthcare) and condensed at 20 mg/ml concentration in a buffer composed with 20 mM Tris-Cl (pH 8.0) and 100 mM NaCl. The protein was crystallized using the sitting drop vapor diffusion method in 0.1M HEPES (pH 8.2), 0.2 M NaCl, and 22.5% polyethylene glycol (PEG) 3350 at 20°C.

### Data Collection, Phasing, and Model Refinement

Diffraction data from the crystals were collected on an in-house Rigaku MM-007HF X-ray source equipped with an R-AXIS HTC image plate detector. The diffraction images were integrated and scaled using HKL2000 [24]. The structure was solved by the molecular replacement method using the program PHASER [25] and a native structure of the PDlim2 PDZ domain (1–82), previously solved in our laboratory (unpublished), as the search model. The structure was built with the program COOT [26] and refined with REFMAC5 [27]. Figures were drawn with the program PYMOL (DeLano Scientific, San Carlos, CA).

## Results

### Identification of PDlim2 as an HN12-NS1 binding protein by yeast two-hybrid screening

In order to search for potential functional targets of the influenza virus NS1 protein, we performed a yeast two-hybrid screen of a human spleen cDNA library using the HN12-NS1 protein as bait. After several rounds of panning, we obtained five positive clones. Sequence analysis of them subsequently identified one of the clones encoding part of the PDlim2. Based on sequence alignments, this clone contains a coding region of 238 amino acid residues, of which residues 1–228 were 100% identical with the N-terminal of PDlim2. PDlim2 is a protein containing both PDZ and LIM domains and is reportedly expressed in epithelial cells and lymphocytes [28], [29]. To date, three isoforms of PDlim2 have been discovered in human cells. The poorly studied isoform 1 has low transcription levels in lung and is expressed only in the cytoskeleton and nucleus [30]. Isoform 3, which is abundant in the heart and brain [30], lacks the LIM domain (the functional region of PDlim2) and is also expressed mainly in the cytoskeleton and nucleus. Previous studies with mouse tissue have indicated that isoform 2 of PDlim2 is transcribed in the spleen, lymphocyte and most highly in the lung [30], [31], which is considered to be the site of infection for influenza virus. Therefore, we aimed to explore the interaction between NS1 and isoform 2 of PDlim2 in all subsequent experiments in this paper.

### Verification of the interaction between HN12-NS1 and PDlim2 via PDZ domain

**Figure 1:**
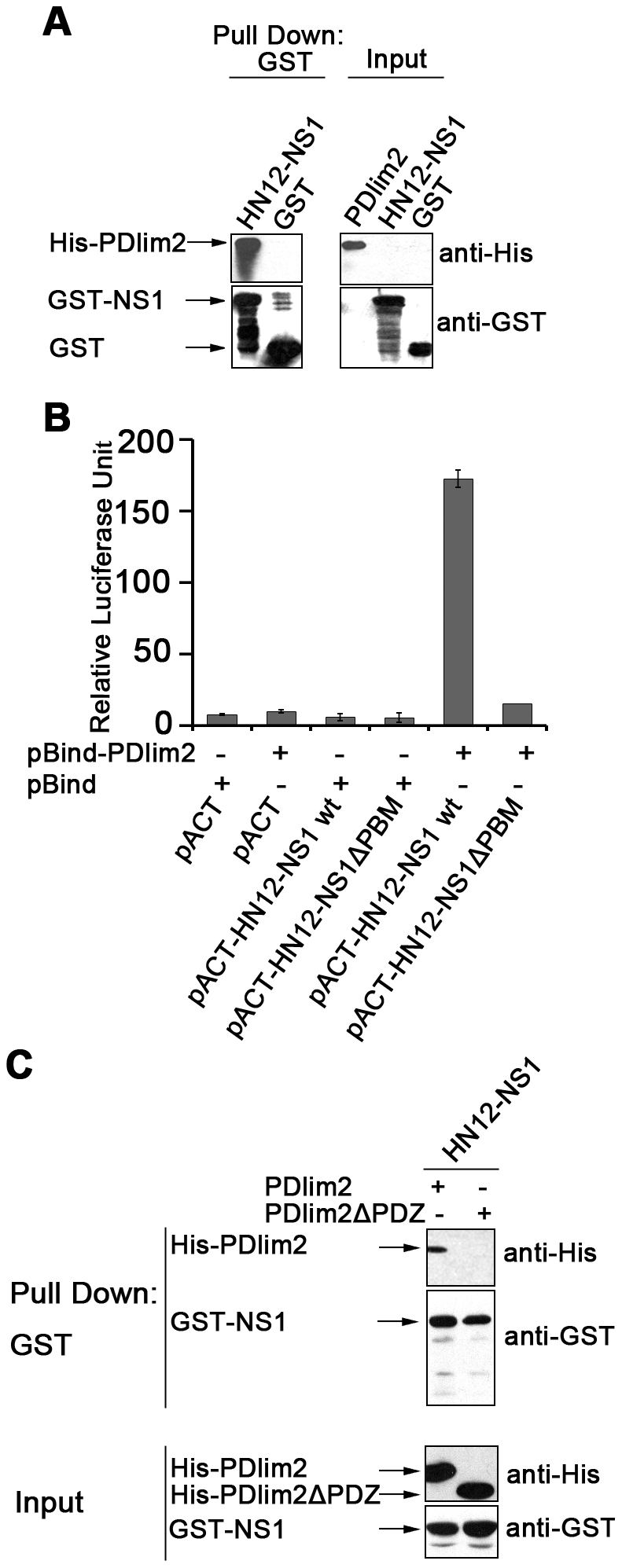
Interaction between PDlim2 and HN12-NS1. (A) GST pull-down assay. Bacterially expressed GST-HN12-NS1 or GST were pre-bound to Glutathione conjugated agarose and then incubated with lysates containing His-PDlim2. The eluates were subjected to SDS-PAGE and Western blot analyses with anti-His or anti-GST antibodies (left), and the input proteins in lysates were also analyzed (right). The arrows indicated the relevant bands. (B) Mammalian two hybrid assay. Reporter plasmid pG5*luc* was co-transfected into HeLa cells with a combination of pACT-or pBind-based constructs as indicated. At 48 h post-transfection, cells were harvested and firefly (from pG5*luc*) and renilla (from pBind) fluorescence unit were measured by luminomitor. The relative luciferase activity (Y-axis) = the unit of firefly luciferase/the unit of Renilla luciferase, and is presented as the mean calculated from three independent experiments. (C) GST pull-down assay for the PDlim2 PDZ domain deletion mutant (PDlim2-ΔPDZ) and HN12-NS1. The experiments were performed as described in (A).

In order to verify the binding result from the yeast two-hybrid screening assay, GST pull-down assays were performed using GST-tagged HN12-NS1 and His-tagged PDlim2. GST-HN12-NS1 or GST was immobilized with Glutathine-conjugated agarose to pull down His-PDlim2. As shown in Fig. 1A, His-PDlim2 can be detected in the precipitation by GST-HN12-NS1, but not by GST. The input of lysate containing His-PDlim2 and the expression of GST or GST-NS1 are shown in the upper panel of Fig. 1A. This binding assay confirms that HN12-NS1 and PDlim2 interact *in vitro*. We also repeated the pull-down assay with a PDlim2 variant in which the PDZ domain was deleted. Figure 1C shows that the wild-type PDlim2 precipitated HN12-NS1 while PDlim2ΔPDZ did not, indicating that the PDZ domain of PDlim2 is required for the interaction with NS1.

To test the intracellular interaction between HN12-NS1 and PDlim2, we adopted a mammalian two hybrid (M2H) assay [32], [33]. PDlim2 and NS1 (or mutations) were cloned into pBind and pACT vectors respectively to generate fusion proteins with the DNA-binding domain (GAL4) or transcription activation domain (VP16). When co-transfected with the reporter plasmid pG5*luc* containing a GAL4 binding site, the positive interactions can be quantified by firefly luciferase in cells. Renilla luciferase was expressed by the pBind vector itself for monitoring the transfection stability. The ratio between firefly and Renilla luciferase activities was used as the relative activity unit (RLU) for the binding. As indicated in Fig. 1B, the reporter luciferase activity reaches 160 RLU (the 5^th^ bar from left) whereas HN12-NS1 lacking PBM exhibits only limited reporter activity (the most right bar) as the negative controls (1–4^th^ bar from left). The mammalian two hybrid analysis demonstrated that the binding of HN12-NS1 to PDlim2 occurred intracellularly via its ESEV sequenced PBM. The deletion of the ESEV PBM of NS1 completely abolished its binding ability with PDlim2.

**Figure 2:**
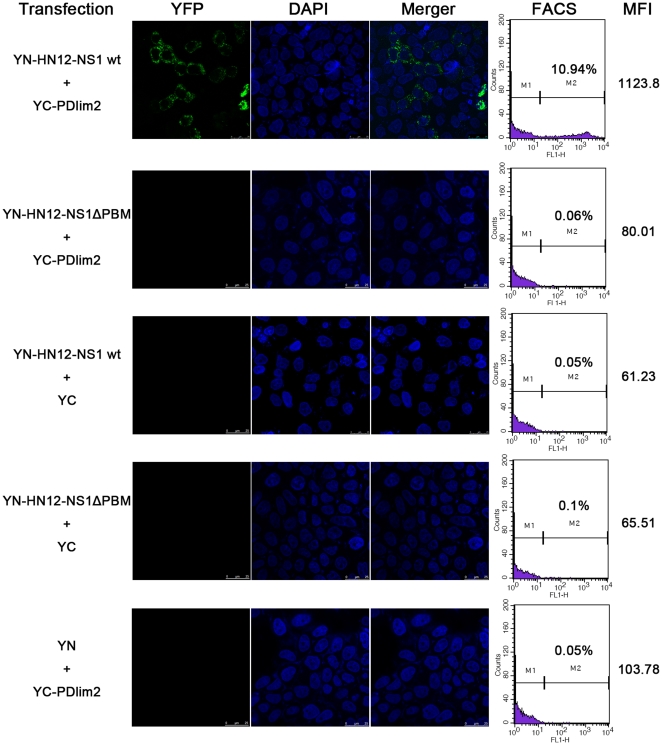
Bifluorescence complementation assay. HeLa cells were co-transfected with constructs encoding the fusion proteins of C- and N-terminal fragments of YFP as indicated. At 24 h post transfection, cultured cells were kept at room temperature for 4 hours and stained by DAPI and detected by Confocal laser scanning microscopy. A portion of each transfected cells were analyzed by fluorescence-activated cell sorting (FACS) with the MFI (mean fluorescence identity) listed in the right column.

To further confirm the interaction *in vivo* and visualize the subcellular localization of the binding, we employed more sensitive bimolecular fluorescence complementation (BiFC) assays [23]. As described in Materials and Methods, we constructed plasmids encoding YN-fused HN12-NS1, namely YN-HN12-NS1 wt, or an NS1 mutant lacking the PBM (YN-HN12-NS1ΔPBM), as well as YC fused with PDlim2 (YC-PDlim2), respectively. The pair of plasmids encoding YN- and YC-fusion proteins were co-transfected into HeLa cells, and the cells were then examined by fluorescence microscopy (Fig. 2, left panels) and analyzed by FACS (Fig. 2, right panels). The interaction indicated by the generation of YFP signals was only observed in cells transfected with full-length wild-type HN12-NS1 and PDlim2 (Fig. 2, top row). An NS1 mutant lacking the PBM exhibited no signal (Fig. 2, 2^nd^ row from the top), indicating that the PBM of NS1 is required for interaction with PDlim2. As expected, the control samples with combinations of the YC or YN fragments and their respective fusion proteins showed barely detectable YFP signals (Fig. 2, the bottom three rows). In addition, the interaction of NS1 with PDlim2 exhibited a punctuate distribution in cytoplasmic space.

The underlying principle of this assay, namely that the N-terminal (YN) and C-terminal (YC) domains of YFP are brought into close proximity to form an intact fluorescent protein once protein binding occurs, allows us to measure the affinity of the binding partners [23]. In this study, the percentage of cells yielding fluorescence and the mean fluorescence intensity (MFI) were determined by FACS analysis, reflecting the binding affinity of HN12-NS1 and PDlim2. As shown in Fig. 2 (the first two panels from the right), 10.94% of cells expressing both HN12-NS1wt and PDlim2 fall into the high fluorescence zone with a mean florescence intensity (MFI) of 1123.8. In comparison, cells expressing an HN12-NS1ΔPBM mutant displayed a low number of cells with negligible YFP signal, as observed in the control groups with expression of only one of the effective binding components. Consistent with the M2H assay results above, the BiFC data strongly demonstrated that the viral HN12-NS1 protein interacts with PDlim2 in the cytoplasm, and that the binding is dependent on the PDZ binding motif of NS1.

### PDlim2 binds specifically to HN12-NS1 but not PR8-NS1

**Figure 3:**
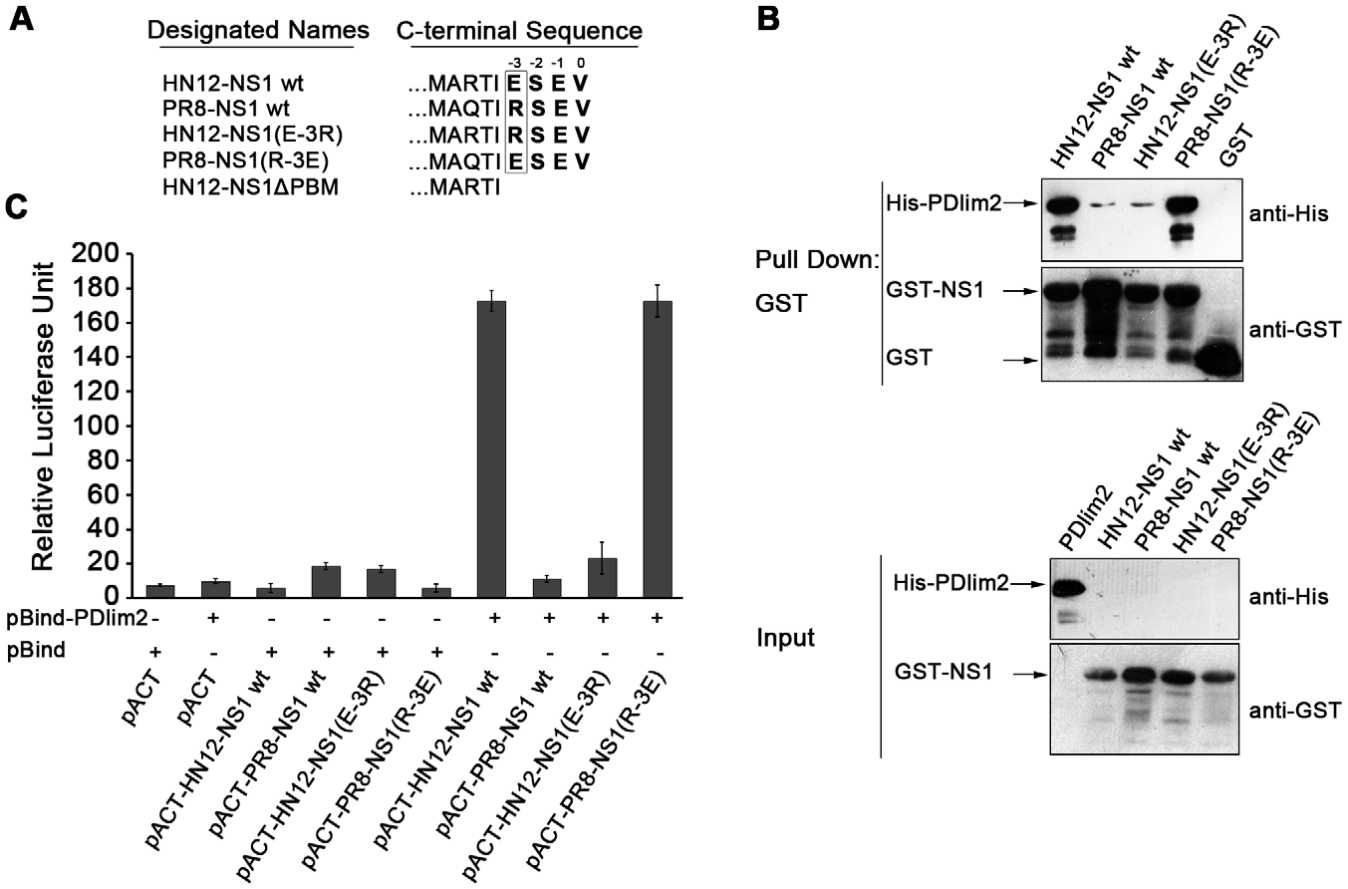
NS1 mutations and PDlim2 binding analyses. (A) Schematic depiction of NS1 mutations. Wild-type NS1 of the H5N1 subtype influenza virus (A/Chicken/Henan/12/2004) is annotated as HN12-NS1wt, and NS1 from the H1N1 subtype (A/Puerto Rico/8/34) as PR8-NS1wt (top two rows). The amino acid in the −3 position of the NS1 PBM was indicated by box. The −3 amino acid swapped to R or E were named as HN12-NS1 (E-3R) or PR8-NS1 (R-3E) respectively (the lower two rows). The PBM deleted mutation of NS1 from the H5N1 subtype was named HN12-NS1ΔPBM (last row). (B) The interaction of PDlim2 with HN12-NS1wt, PR8-NS1wt, HN12-NS1 (E-3R) or PR8-NS1 (R-3E) was analyzed by GST-pull down assays, as described in Experimental Procedures, with GST alone as a control. Elution (top panel) and the amount of input lysate (bottom panel) were performed by SDS-PAGE and Western blot. The arrows indicated the relevant bands after detected by anti-His or anti-GST antibodies. (C) Mammalian two hybrid assay. NS1 or its mutations and PDlim2 were constructed into pACT and pBind, respectively. This pair of plasmids, together with pG5*luc*, were transfected into HeLa cells. The relative luciferase unit of cell lysates was quantified by the Dual-Luciferase Reporter Assay System (Promega). The data represent three independent experiments.

The C-terminal PBM of the NS1 protein differs in sequence between influenza virus strains, and the NS1 protein with ESEV PBM as commonly found in H5N1 avian influenza viruses is linked with high pathogenicity [10]. As the only difference between the two PBMs is the amino acid residue in the −3 position, we therefore “swapped” the PBMs of HN12-NS1 wt and PR8-NS1 wt by simply mutating the −3 position Arg of PR8-NS1 to a Glu, designated PR8-NS1 (R-3E), and the E (-3) of HN12-NS1 to Arg, designated HN12-NS1 (E-3R), as shown in Fig. 3A. GST pull-down assays using these constructs showed that the HN12-NS1 (E-3R) mutant lost its binding ability to PDlim2 (Fig. 3B, lane 3), whereas the PR8-NS1 (R-3E) mutant acquired binding ability (Fig. 3B, lane 4) comparable to that of the wild-type GST-HN12-NS1 wt (Fig. 3B, lane 1). The pull-down by GST alone was used as a negative control. It was noted that a trace amount of NS1 was detected in the pull-down assays with wild-type PR8-NS1 and PR8-NS1 (R-3E) (Lanes 2 and 3). Considering the comparable amounts of input in each pull-down assay, the association of wild-type PR8-NS1 with PDlim2 was not as favorable as wild-type HN12-NS1wt. The mammalian two-hybrid assays further validated the selectivity of PDlim2 for a Glu residue in the −3 position, since replacing the −3 Arg with Glu raised the activity of the reporter promoter to the same level as wild-type HN12-NS1 (Fig. 3C).

The above *in vitro* and *in vivo* assays involving PBM-swapping mutations in NS1 demonstrated that the −3 position amino acid residue is a critical site for the binding selectivity of PDlim2.

### Structure Determination and Refinement

In order to investigate the mechanism of the interaction between HN12-NS1 and PDlim2, we first crystallized the native form of the PDlim2 PDZ domain [34] and chemically synthesized a hexapeptide (sequence: TIESEV) derived from the C-terminal of HN12-NS1. Unfortunately, however, we failed to successfully crystallize the PDZ-peptide complex either by soaking or co-crystallization. Therefore, following a strategy employed for solving PDZ-peptide complex structures in several other studies [19], [20], we fused the PDZ domain (1–83) of PDlim2 and the C-terminal hexapeptide of HN12-NS1 together, as described in Materials and Methods. This approach yielded crystals in the space group I23 with unit cell parameters a = b = c = 81.9 Å, α = β = γ = 90°. The complex structure was solved at 2.2 Å resolution using the molecular replacement method. Refinement of the fusion protein structure resulted in a final model with crystallographic R-factor (R_cryst_) of 0.200 and a free R-factor (R_free_) of 0.249. Crystallographic statistics are summarized in Table 1 and Table 2.

**Table 1:** Data collection statistics.

Parameters	Statistics
Space group	I23
Unit cell parameters	a = b = c = 81.9 Å, α = β = γ = 90°
Wavelength (Å)	1.5418
Resolution (Å)	2.20
Measured reflections	49704
Unique reflections	4769
Completeness (%)	99.8 (100)^a^
R_merge_ b	0.062 (0.374)
Redundancy	10.4 (10.4)
I/σI	32.6 (6.2)

a Numbers in brackets refer to the statistical data for the outer resolution shell.

b R_merge_ = Σ_h_Σ_i_|I_i_ (h)−<I (h)>|/Σ_h_Σ_i_<I_i_ (h)>, where I_i_ (h) is the intensity of an individual measurement of the reflection, and <I_i_ (h)> is the mean intensity of the reflection.

**Table 2:** Structure refinement statistics.

Parameters	Statistics
R_cryst_ a	0.200
R_free_ b	0.249
Bond rms deviation (Å)	0.008
Angle rms deviation (°)	1.187
Average B factor (Å^2^)	27.5
Ramachandran plot (%)^c^	98.81/1.19/0

a R_cryst_ = Σ (||F_obs_|−|F_calc_||) / Σ|F_obs_|, where F_obs_ and F_calc_ are the observed and calculated structure-factor amplitudes, respectively.

b R_free_ was calculated as R_cryst_ using the reflections in a test set not used for structure refinement, which is a randomly selected subset containing 4.5% of unique reflections.

c Calculated using MolProbity. Numbers reflect the percentage of residues in the preferred, allowed, and disallowed regions, respectively.

### Structure of the PDlim2 PDZ domain in complex with the C-terminal hexapeptide of HN12-NS1

Each asymmetric unit contains one fusion protein with 89 amino acid residues, 47 water molecules and 1 sodium ion. The final model has good stereochemistry with no amino acid residues located in disallowed regions of the Ramachandran plot. All side chains were assigned with the exception of Arg53 and Arg77, which were not placed due to insufficient electron density. Experimental structure factors and the coordinates of the final refined model have been deposited in the Protein Data Bank (PDB) with accession number 3PDV.

**Figure 4:**
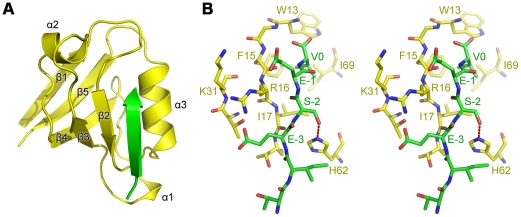
Structure of the PDlim2 PDZ domain in complex with the HN12-NS1 C-terminal hexapeptide extension. (A) Ribbon diagram of the PDlim2 PDZ domain (yellow) is from one PDZ-PBM fusion protein. The PBM peptide (green) presented here is from a neighboring PBM-PDZ fusion protein. (B) Stereo view of the interaction site. Carbon atoms from the PDlim2 PDZ domain and HN12-NS1 C-terminal hexapeptide extension are colored yellow and green respectively. Nitrogen and oxygen atoms are colored blue and red, respectively. Hydrogen bonds are indicated by red dashed lines.

The structure of the PDZ region (1–83) of PDlim2 is consistent with a classic type I PDZ domain composed of five β-strands (β1–β5) and three α-helices (α1, α2 and α3), as shown in Fig. 4A. The five β-strands form an anti-parallel β-sheet with a topological order of β1-β5-β4-β3-β2. The small α1 helix is localized between β2 and β3, while α2 lies between β3 and β4, and α3 is situated between β4 and β5. The three α-helices cap the two open ends of the partially open β-sheet barrel, with α1 and α3 at the β2 end and α2 at the β1 end. The interaction site of the PDZ domain is occupied by the C-terminal HN12-NS1 hexapeptide extension from a neighbouring PDZ-peptide fusion protein (Fig. 4A and Fig. S1A).

The detailed mode of interaction between the PDZ domain of PDlim2 and the C-terminal NS1 hexapeptide extension is consistent with the classical type I PDZ domain-ligand peptide interaction mode exhibited by other PDZ-ligand structures solved using the co-crystallization strategy [35] or the PDZ-ligand fusion strategy [19], [20] as employed in this study (Fig. 4B and Fig. S1B). Five residues in the C-terminal HN12-NS1 extension peptide (from positions 0 to −5) form a β-strand that pairs with strand β2 of the PDZ domain in an anti-parallel manner. In the PDlim2 PDZ domain, the hydrophobic side chains of residues Phe15 and Ile17 from β2, Ile69 from α3, and the side chain of Trp13 compose a hydrophobic pocket which facilitates packing of the side chain of the valine residue in position 0 of the HN12-NS1 extension. The residue in the −2 position of the NS1 extension is recognized by residue His62 (from α3) of the PDZ domain by a hydrogen bond between Ser223-Oγ and His62-Nε, with a distance of 2.6 Å. The two glutamate residues at positions −1 and −3 of the NS1 extension are stabilized via salt bridges with the positively charged PDZ residues Arg16 and Lys31. No other obvious interactions can be observed between the NS1 extension peptide and the PDZ domain.

### Characterization of the roles of Arg16 and Lys31 in PDlim2

**Figure 5:**
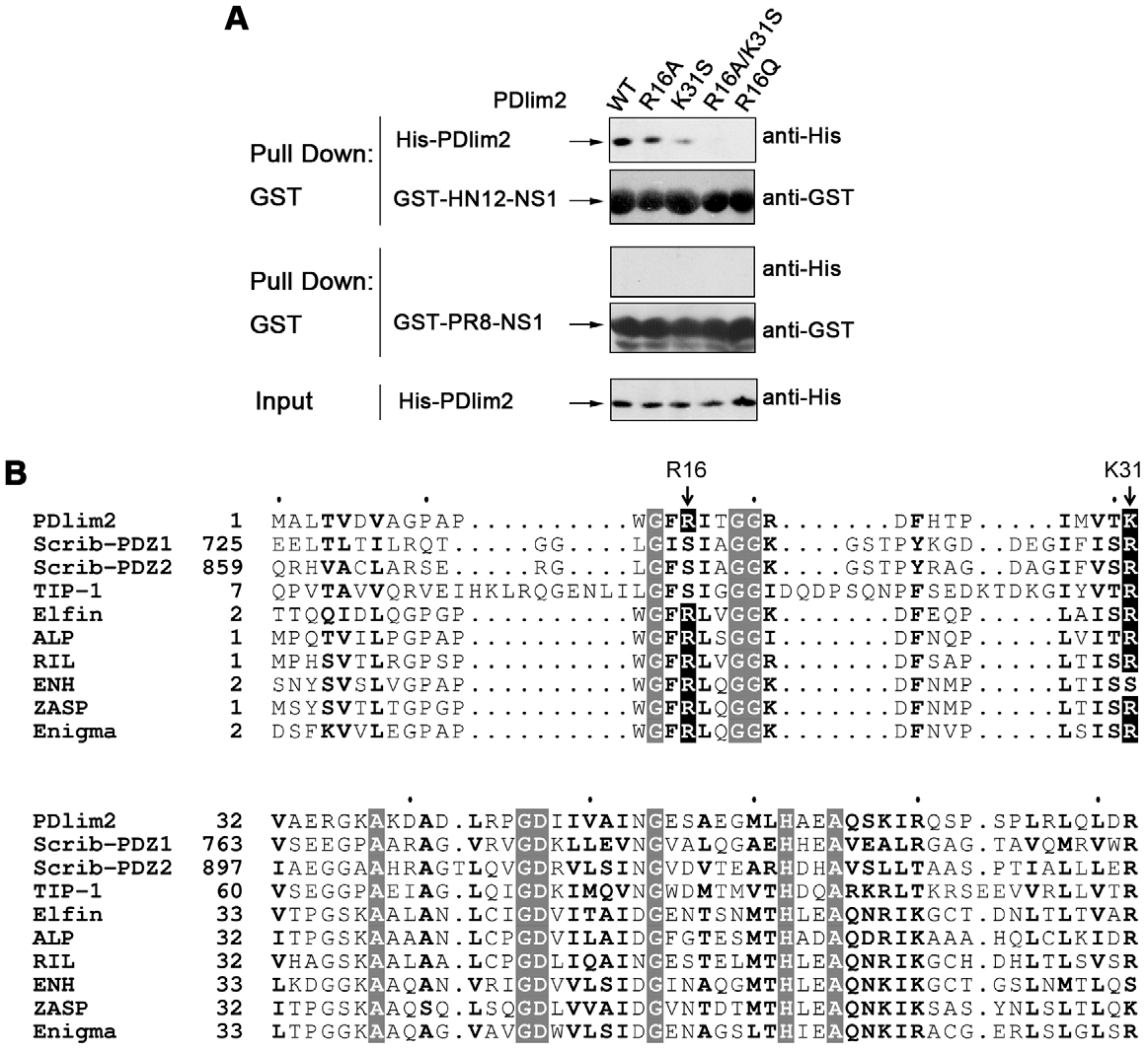
Analysis of the binding interface between PDlim2 and HN12-NS1. (A) GST pull-down assay. PDlim2 and its mutations were His-tagged and expressed in *E. coli*. GST- HN12-NS1 or PR8-NS1 were linked to GST agarose beads, which were added to *E. coli* expressed His-PDlim2 or its mutations and incubated for 2 h. The pellets were washed and eluted by boiling. The elution was then subjected to SDS-PAGE and Western blot analysis. (B) Sequence alignment of PDZ domains from different proteins. Scrib-PDZ1 and Scrib-PDZ2 refer to the first and second PDZ domain of Scribble. Conserved residues are written in bold letters, and the identical ones are highlighted with gray background. Basic residues equivalent to Arg16 or Lys31 of PDlim2 are highlighted with black background and the sites are indicated with arrows. Sequences were aligned by the ClustalX program, and the alignment was drawn using the online ESPript server (http://espript.ibcp.fr/).

To address the importance of the Arg16 and Lys31 residues of PDlim2 in the interaction between its PDZ domain and HN12-NS1, we constructed a series of PDlim2 mutations at these two positions in the PDZ domain and examined their binding ability with HN12-NS1 or PR8-NS1 by GST pull-down assays, as described previously (Fig. 5A). The wild-type PDlim2 (WT) showed the strongest binding to HN12-NS1 (Fig. 5A, lane 1), while the R16A and K31S mutants exhibited reduced affinity with HN12-NS1 (Fig. 5A, lane 2 and 3). Furthermore, the ability of the PDlim2 R16A/K31S double mutant to associate with HN12-NS1 was completely abolished (Fig. 5A, lane 4), reflecting the disruption of the salt bridges. Therefore, either one of the basic residues (Arg16 or Lys31) would be sufficient to stabilize the interaction between PDlim2 and HN12-NS1 via charge interactions, or the availability of both positively charged residues would enhance the binding. It is likely that the contribution of Lys31 to the interaction between PDlim2 and HN12-NS1 is much greater than Arg16, as the amount of the R16A mutant PDlim2 protein pulled down by HN12-NS1 was higher than the K31S mutant (Fig. 5A, lane 2 and 3).

The higher binding of HN12-NS1 by the R16A PDlim2 mutant than by the K31S mutant led us to assume that the R16A mutation may provide a favorable advantage to the binding, despite its charge. We therefore mutated Arg16 to a glutamine residue, which also features a long side chain but is charge free, and examined its binding affinity to HN12-NS1. The results showed that the R16Q mutant was unable to bind with the NS1 protein, even though Lys31 was still present in the mutant PDlim2 protein (Fig. 5A, lane 5). These observations imply that Arg16, in addition to playing a positive role in providing salt bridges for binding HN12-NS1, may also impose a steric requirement on the PBM binding. In wild-type PDlim2, the positive effect of Arg16 is dominant. Lys31 is not as close to the two glutamine residues in the ESEV PBM (Fig. 4B) and should inflict little or no steric influence, therefore contributing more than the bi-functional Arg16 to the interaction between PDlim2 and HN12-NS1.

As shown in Fig. 5B, a sequence alignment of PDlim2 with selected PDZ domain proteins predicted to bind to the ESEV PBM of NS1 reveals that all except TIP-1 and the first two PDZ domains of Scribble have a conserved arginine residue equivalent to Arg16 of PDlim2. A conserved arginine residue can also be found in an equivalent position to the basic residue Lys31 of PDlim2 in all other PDZ domains, with the exception of ENH.

### Interaction of NS1 and PDlim2 may not affect NF-κB activity, nor STAT1 mediated signal transduction -

**Figure 6:**
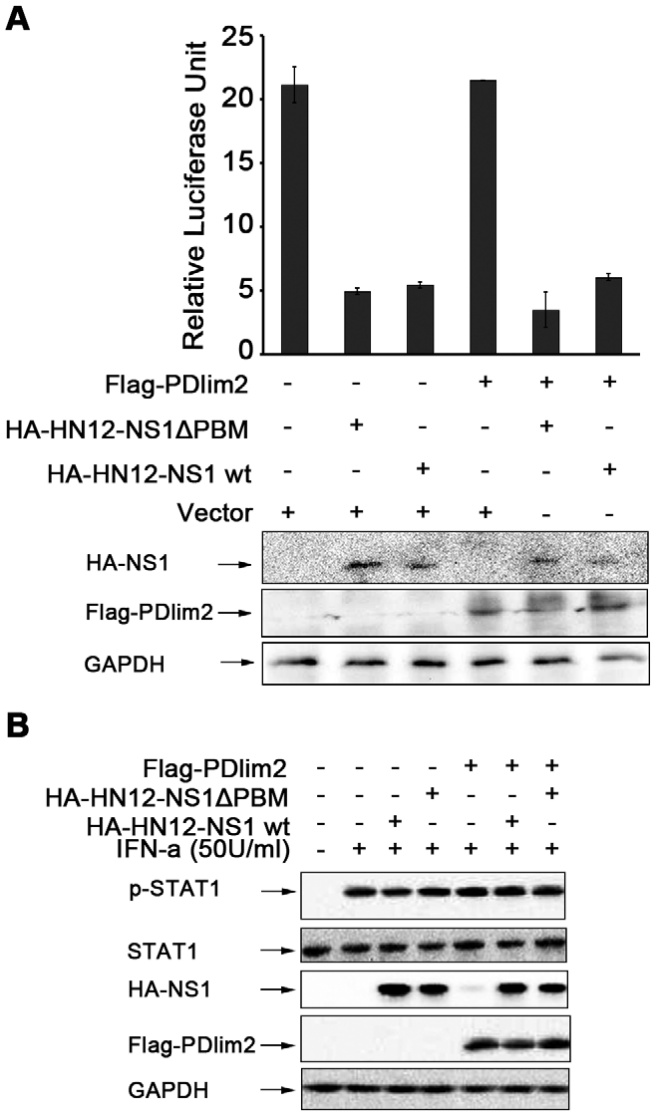
Effects of the PDlim2 and HN12-NS1 interaction on NF-κB activity or STAT1 phosphorylation. (A) HeLa cells was transfected with the reporter plasmid pNF-κB-luc and ptk-RL, together with the indicated plasmids. After 24-hour post-transfection, cells were stimulated by LPS (10 µg/ml) for 5 hours. Cells were harvested and firefly and renilla fluorescence unit were measured by luminomitor. The relative luciferase activity (Y-axis) = the unit of firefly luciferase activity / the unit of Renilla luciferase activity (to indicat transfection efficiency, and is presented as the mean calculated from three independent experiments. The expression of NS1 and PDlim2 were analyzed by Western blot with indicated antibodies (lower panels). (B) HeLa cells were transfected with the combination of the plasmids as indicated at the top of the figure and treated by IFN-α at 50 U/ml for 4 hours. The cell lysates were analyzed by SDS-PAGE and Western blot, with antibodies specific for phosphorylated STAT1, STAT1, HA-tag, Flag-tag, and GAPDH as loading control.

The discovery of PDlim2 as a new cellular target for HN12-NS1 prompted us to investigate the biological significance of this selective interaction and the mechanism involved in the virulence of H5N1 influenza viruses. Takashi and colleagues previously reported that PDlim2 has E3 ligase activity for the p65 subunit of NF-κB and controls the transcription activity of NF-κB via the ubiquitination and degradation of p65 [36]. In order to verify whether NS1 participates in this pathway, we established an NF-κB reporter assay in HeLa cells co-transfected with plasmids encoding PDlim2 and wild-type HN12-NS1 or the HN12-NS1ΔPBM mutant. When induced by lipopolysaccharides (LPS), the PDlim2 transfected group showed no inhibition of the reporter activity (Fig. 6A, lane 4 from the left) in this assay. As reported in a previous study [3], both the wild-type HN12-NS1 and the HN12-NS1ΔPBM mutant transfected groups could suppress the reporter activity [3] (Fig. 6A, lane 2 and 3 from the left), but there was no significant difference between them, even if PDlim2 was co-transfected or not (Fig. 6A, lane 5 and 6 compared with lane 2 and 3). The expression levels of PDlim2 and NS1 detected by Western blot indicated the homogeneity of transfection in each group. This result implies that the binding between PDlim2 and HN12-NS1 may not influence the activity of NF-κB.

It was also reported that PDlim2 can impact the tyrosine phosphorylation of STAT4 or lead to degradation of STAT1 and STAT4 under stimulation [31]. We also examined whether HN12-NS1 can affect this PDlim2-STAT1 pathway by co-transfecting PDlim2 with wild type HN12-NS1 or the HN12-NS1ΔPBM mutant into HeLa cells. The stimulation of IFN-α induced a strong phosphorylation of the endogenous STAT1 compared with the un-stimulated sample, as indicated in Fig. 6B. However, compared to previously reported data [7], [18], the levels of STAT1 phosphorylation in our experiments were similar in the presence of wild-type HN12-NS1 and HN12-NS1ΔPBM in combination with PDlim2. These results suggest that the interaction between NS1 and STAT1 may be complicated and further studies are required to clarify the function of the interaction between NS1 and PDlim2 in interferon-induced STAT1 signaling.

## Discussion

The NS1 PBM bearing the sequence ESEV (NS1-ESEV) has been found in highly virulent human virus strains from the 2003–2004 outbreak in Hong Kong, Vietnam, and Thailand, while NS1 PBM bearing the sequence RSKV (NS1-RSKV) were usually found in milder human viruses [10]. Intensive studies have demonstrated the multiple roles of influenza virus NS1 in anti-host immune responses, such as interfering in RIG-I pathways to interrupt interferon production [4], and inhibiting PKR and OAS [5], [6] to halt host protein synthesis. The mechanisms that distinguish the HPAI NS1 PDZ binding motifs (ESEV) from the other human influenza virus strains (RSEV or RSKV) were not understood until the interaction between NS1 and Scribble was reported to regulate cell apoptosis during influenza virus infection. In a recent study, the PDZ domain protein Scribble was demonstrated to selectively interact with NS1 bearing an ESEV PBM via its first two PDZ domains [37]. Other PDZ domain proteins including MAGI-1, -2, -3 and Dlg-1 were also found to interact with NS1 proteins bearing ESEV-like PBM [18], [37]. In this report, we performed yeast two-hybrid screening, originally aiming to find potential target(s) of Avian Influenza virus NS1 that functions in the immune responses. Considering that human spleen is rich in macrophage, antigen presenting cells, and lymphocytes, we therefore choose human spleen cDNA library and using full-length wild type HN12-NS1 and a human PR8-NS1 as baits respectively, rather than using ESEV or RSKV peptides as probes [10]. Of the five candidate clones, though ubiquitously expressed in other tissues as well, PDlim2 is the only one that is highly expressed in lungs [30], [31]. Interestingly, PDlim2 contains a PDZ domain and exhibits PBM selectivity towards HN12-NS1 (ESEV) over PR8-NS1 (RSEV), leading us to speculate that PDlim2 is a possible cellular target for HN12-NS1 (ESEV). Further investigation is ongoing to understand the biological relevance of the interaction between PDlim2 and HN12-NS1 (ESEV).

PDZ domains exist in numerous proteins as structural scaffolds, linking the biological activities of the other functional domains. PDlim2 recruits p65 of NF-κB to the Lim ubiquitin ligase activity [36]. The NS1 proteins of many HPAI strains differ from those isolated from milder strains by a single amino acid in their canonical PDZ binding motifs. We established that PDlim2 is able to distinguish NS1 proteins from different influenza virus strains and binds selectively to the NS1 protein encoded by a highly pathogenic H5N1 avian influenza virus, HN12-NS1. As PDlim2 contains only one PDZ domain, it can be considered as a good model to investigate the binding mechanism between PDZ domains and the NS1 PBM. Our crystal structure reveals that the charge interactions between PDlim2 and HN12-NS1 are crucial for their binding interaction. In PR8-NS1, the −3 position amino acid residue is an arginine, a positively charged residue with a long side chain. This residue is expected to be unable to form a salt bridge with either of the two basic residues (Arg16 and Lys31) in the PDlim2 PDZ domain. Furthermore, the arginine residue in the −3 position of the PR8-NS1 PBM can inflict strong charge repulsion with Arg16 and Lys31 in the PDZ domain of PDlim2, severely compromising the binding between the RSEV PBM and the PDlim2 PDZ domain. The K31S or R16A mutants of PDlim2, which already preclude the steric influence caused by Arg16, also exhibit no binding affinity with PR8-NS1 (Fig. 5A). Although the −1 position glutamate residue in RSEV should have been able to form salt bridges with Arg16 or Lys31, the charge repulsion between the −3 position arginine residue of the RSEV PBM and either the Arg16 or Lys31 residue of PDlim2 was implied to be sufficient to disrupt the PDlim2-NS1 interaction. The PDlim2 R16A/K31S double mutant, which failed to stabilize residues in the −1 and −3 positions in the ESEV PBM, should also be unable to stabilize residues in the −1 and −3 positions in the RSEV PBM, which is consistent with the lack of binding observed with PR8-NS1 (Fig. 5A, lane 4).

In human influenza viruses, NS1 proteins bearing RSKV or KSEV PBM have also been identified [10]. In the case of PDlim2, the RSKV PBM contains not only the −3 position arginine residue, but also another basic amino acid in the −1 position. Based on our above analysis, it is reasonable to consider that this PBM should be unable to bind with the PDZ domain of PDlim2. As we were able to detect only trace amounts of wild-type PR8-NS1 binding with PDlim2, the RSKV PBM would be expected to have even lower affinity than the RSEV PBM. In the case of the KSEV PBM, the residue in the −3 position is positively charged and should exhibit slightly higher affinity with PDlim2 than the RSEV PBM, although the interaction would still be considerably weaker than for the ESEV PBM. Although the arginine residue in the −3 position has a side chain even longer than that of lysine, the Cδ, Nε, Cζ, Nη1, and Nη2 atoms must lie in the same plane, and thus its flexibility is considerably lower than the side chain of lysine. When interacting with the PDZ domain, the flexible lysine residue in KSEV may be driven to a position that minimizes the charge repulsion. Based on the above analyses, we would expect to rank the NS1 PBM in terms of their binding abilities to PDlim2 as follows: ESEV>KSEV>RSEV>RSKV. In studies by Jackson and colleagues, recombinant influenza viruses differing only in their NS1 PBMs were used to infect mice and the mean lethal dose (MLD_50_) was measured. The virulence of the viruses, ranked in decreasing order of MLD_50_ (from large to small), were viruses bearing the ESEV, EPEV, KSEV, and RSKV PBM [14]. A recombinant virus in which the PBM was truncated showed the lowest virulence. Interestingly, the virulence of the recombinant viruses bearing the above PBMs is consistent with the binding abilities of those PBMs to PDlim2, as proposed above. EPEV is not a typical PDZ binding motif, although it has been reported to bind with certain PDZ domains, such as the PDZ domain of Dsh [10]. As EPEV also contains the same two acidic glutamate residues as ESEV, we speculate that these two glutamates in EPEV should also be able to interact with the two basic residues in the PDZ domain, although the proline residue in the −2 position is expected to alter the conformation of the PBM and subsequently reduce the affinity of the interaction. Jackson and colleagues further showed that human influenza viruses containing the three most virulent NS1 PBMs (ESEV, EPEV, and KSEV) correspond to the highly virulent strains discovered from the 2003–2004 outbreak in Hong Kong, Vietnam, and Thailand; the 1997–1999 outbreaks in Hong Kong; and the 1918 “Spanish flu” outbreak, respectively [10]. In contrast, influenza viruses containing the RSEV or RSKV PBMs, which were speculated to exhibit barely detectable binding with PDlim2, have not been associated with large influenza outbreaks nor with high pathogenicity. However, in other studies, evidence has indicated that there were minor differences between the ESEV, RSEV or truncated PBM NS1 proteins in the control of its nuclear migration and mean lethal dose in infected cells [16]; the human C-terminal RSKV PBM was found to increase viral replication in human and ducks, while the avian C-terminal ESEV PBM increased virulence in mice [15]. Furthermore, the nucleic acid region of the influenza virus that encodes the PBM of NS1 also participates in encoding another protein, NEP, via a different reading frame [14]. The above findings suggest that the relationship between the sequence variation in the PBM and the virulence of influenza virus is complicated and requires further investigation. The selective binding by NS1 of PDZ containing proteins, such as PDlim2, may provide a key starting point for future studies of the pathogenic mechanism and virulence determining factors of influenza viruses.

During the course of analysis, a protein called Scribble containing four PDZ domains was reported to be involved in an NS1-mediated anti-apoptosis process during influenza virus infection. This protein was also reported to bind with only the ESEV PBM, but not the RSKV PBM, via its first two PDZ domains [17]. We compared the sequences of the two PDZ domains of Scribble with the PDZ domain of PDlim2 and observed that each of the first two PDZ domains of Scribble has a basic residue equivalent to Lys31 of PDlim2 (Fig. 5B). Although each of the first two Scribble PDZ domains lacks a basic residue equivalent to Arg16 of PDlim2, the Ser residues may allow the special selectivity to the NS1 (ESEV). In the study by Obenauer and colleagues, TIP-1 was also in the list of potential ESEV PBM binding proteins [10]. Comparing the structures of TIP-1 [35] and our structure of the PDlim2 PDZ domain, TIP-1 also contains an equivalent basic residue (Arg59) to the Lys31 residue of PDlim2, but lacks the other basic residue equivalent to the Arg16 residue of PDlim2. Thus, TIP-1 could be considered as another “PDlim2-like” PDZ domain containing protein. Besides PDlim2, there are a series of human PDZ and LIM domain containing proteins, including actinin-associated LIM protein (ALP, PDlim3), Elfin (CLP36, PDlim1), Enigma (LMP-1, PDlim7), Enigma homologue (ENH, PDlim5), reversion-induced LIM protein (RIL, PDlim4), and Z-band associated protein (ZASP, Cypher, Oracle, PDlim6) [38]. A sequence alignment revealed that the equivalent residue to Arg16 of PDlim2 is strictly conserved in all of these proteins (Fig. 5B). Theoretically, these proteins should favor ESEV PBM over RSEV or RSKV PBM, and thus be possible targets of HPAI NS1. However, the physiological association should also be attested for deciphering the role of the NS1 PBM in determining the virulence of the influenza virus.

The importance of PDlim2 in host innate immune responses has been recognized recently but not fully investigated. A study by Tanaka and colleagues revealed the significant roles of PDlim2, as an E3 ubiquitin ligase, in terminating NF-κB activation through intranuclear sequestration and subsequent degradation [36]. However, in our reporter assay, equal suppression of NF-κB was seen in cells transfected with HN12-NS1wt and the HN12-NS1ΔPBM mutant (Fig. 6A), or in cells infected with influenza viruses lacking the ESEV PBM [39]. Thus, it is likely that the effect on NF-κB by NS1 is independent of its PDZ binding.

Transfected NS1 was reported to downregulate the phosphorylation of STAT1 under IFN stimulation [7], [18]. However, in all of our repeated experiments, wild-type HN12-NS1 did not exhibit a marked impact on the IFN-induced phosphorylation of STAT-1 (Fig. 6B). We cannot rule out the possibility that the effect of the NS1-PDlim2 interaction on interferon-STAT1 signaling is strain-specific, as the NS1 of the influenza A virus we employed is different from the strains used in other studies [7], [18]. Although close inspection shows that a slightly lower level of STAT-1 phosphorylation could be detected in the wild-type HN12-NS1 transfected group (Fig. 6B, lane 3), it is not evident enough to be used as a readout. In addition, transfected NS1 may behave differently from the NS1 in cells infected with influenza virus or recombinant variants by reverse genetic modifications [40]. Due to stringent biological safety restrictions in mainland China, we were unable to test with recombinant viruses containing a H5N1 NS1 fragment. The discrepancy between transiently expressed NS1 and NS1 in infected cells may be more-or-less attributed to the output.

We reported here the structural characteristics of NS1 PBM with a PDZ-containing protein, PDlim2, and this feature and selectivity may be applied to a broader range of PDZ-containing target for the NS1 in Avian influenza virus. The biological effects of NS1 (ESEV) mediated by PDlim2 are to be explored along with a further understanding of the function of PDlim2, as well as efficient and available approaches to analyze the effects of NS1 in more physiological circumstances.

## Supporting Information

Figure S1 **Structure of the PDlim2 PDZ domain fused with the HN12-NS1 C-terminal hexapeptide, and the comparison with similar structures.** (A) Ribbon diagram of two adjacent PDlim2 PDZ-hexapeptide fusion proteins. (B) Superposition of PDZ-ligand complex structures. The structure of PDlim2 PDZ domain in complex with its HN12-NS1 C-terminal hexapeptide ligand is shown in yellow. The structure of the first PDZ domain of the Na^+^/H^+^ exchanger regulatory factor in complex with a pentapeptide ligand from the carboxyl-terminal of the β2 adrenergic receptor (PDB code: 1GQ4) or cystic fibrosis transmembrane conductance regulator (PDB code: 1I92) or platelet-derived growth factor receptor (PDB code: 1GQ5) is showed in red, green, or blue, respectively. The structure of TIP-1 in complex with c-terminal hexapeptide of Kir2.3 is shown in magentas. Secondary structures are only assigned to the ligand peptides.
(TIF)
