# Supplementary figures and images for: PDlim2 Selectively Interacts with the PDZ Binding Motif of Highly Pathogenic Avian H5N1 Influenza A Virus NS1
Source: PLoS One. 2011 May 23;6(5):e19511. doi: 10.1371/journal.pone.0019511 (PMC3100292; doi:10.1371/journal.pone.0019511)

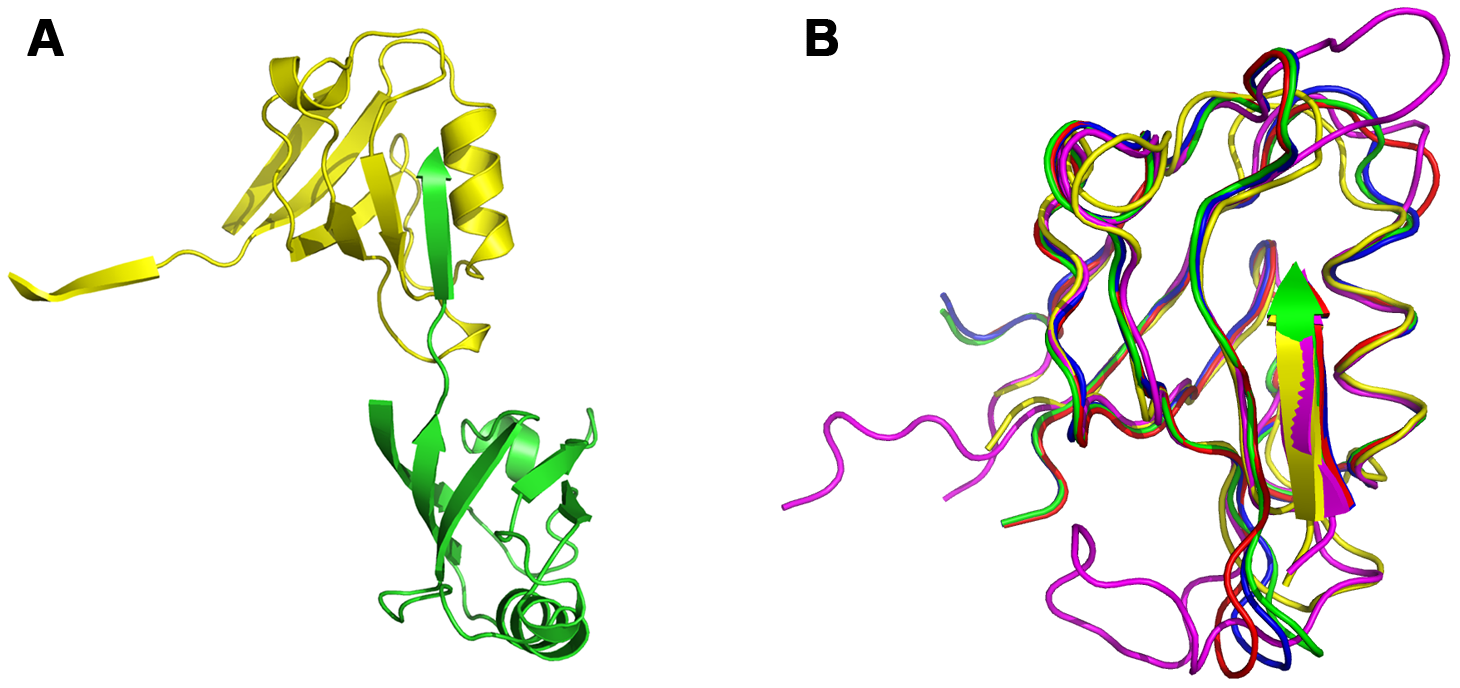

Supplement: Figure S1 — Structure of the PDlim2 PDZ domain fused with the HN12-NS1 C-terminal hexapeptide, and the comparison with similar structures. (A) Ribbon diagram of two adjacent PDlim2 PDZ-hexapeptide fusion proteins. (B) Superposition of PDZ-ligand complex structures. The structure of PDlim2 PDZ domain in complex with its HN12-NS1 C-terminal hexapeptide ligand is shown in yellow. The structure of the first PDZ domain of the Na+/H+ exchanger regulatory factor in complex with a pentapeptide ligand from the carboxyl-terminal of the β2 adrenergic receptor (PDB code: 1GQ4) or cystic fibrosis transmembrane conductance regulator (PDB code: 1I92) or platelet-derived growth factor receptor (PDB code: 1GQ5) is showed in red, green, or blue, respectively. The structure of TIP-1 in complex with c-terminal hexapeptide of Kir2.3 is shown in magentas. Secondary structures are only assigned to the ligand peptides. (TIF) [file pone.0019511.s001.tif]
